# The Changing Concepts of Vesicoureteral Reflux in Children

**DOI:** 10.1155/2008/767138

**Published:** 2009-03-10

**Authors:** Walid A. Farhat, Hiep T. Nguyen

**Affiliations:** ^1^The Hospital for Sick Children, 555 University Avenue, Toronto, Ontario, Canada M5G 1X8; ^2^Children's Hospital Boston, 300 Longwood Avenue, Boston, MA 02115, USA

Over the past two decades, tremendous progress has been made in the diagnosis, management, and treatment of vesicoureteral reflux (VUR), thus changing our understanding of this entity from being surgical to medical disease.

VUR is most often identified following investigation for other urinary tract problems such as urinary tract infection (UTI) and prenatal hydronephrosis or in evaluation of a family history of VUR. The prevalence of VUR ranges from 0.4% to 1.8% of asymptomatic children but increases to 30–50% in children with a history of a febrile UTI. VUR in the presence of a UTI can lead to pyelonephritis and renal injury with permanent scarring (reflux nephropathy). Reflux nephropathy remains an important cause of renal failure in children and the subsequent need for renal transplantation in the United States. Furthermore, it is evident that VUR may be a component of dysfunctional lower urinary tract (i.e., dysfunctional elimination syndrome) and thus has further enhanced our understanding of this entity.

Since VUR may resolve spontaneously in the majority of patients without requiring surgical intervention, children with VUR are traditionally managed with antibiotic prophylaxis with the primary goal of preventing the long-term complications associated with VUR such as renal scarring, hypertension, and renal insufficiency/failure by the prevention of urinary tract infection. However, surgical correction may be required if there is a break-through UTI or failure to resolve after a period of observation. More recently, the management and rationale for the treatment of VUR have been re-evaluated. The risks and benefits of diagnosing VUR are being questioned from a health impact and financial level. For instance, the efficiency of prophylactic antibiotic in the management of VUR is being challenged. Furthermore, alternative and less invasive methods of treating VUR are being proposed with undefined long-term outcomes. Consequently, many controversies now exist for the management of VUR.

In this special issue, we have assembled 23 articles addressing the controversies associated with the diagnosis, management, and treatment of VUR. We hope to provide some understanding of what we know and do not know about VUR and stimulate scientific evaluations of VUR and its management.

*Walid A. Farhat* *Hiep T. Nguyen*

